# Intraoperative robotic-assisted low anterior rectal resection performance assessment using procedure-specific binary metrics and a global rating scale

**DOI:** 10.1093/bjsopen/zrac041

**Published:** 2022-05-11

**Authors:** Marcos Gómez Ruiz, Samson Tou, Anthony G. Gallagher, Carmen Cagigas Fernández, Lidia Cristobal Poch, Klaus E. Matzel

**Affiliations:** 1 Colorectal Surgery Unit, General Surgery Department, Marqués de Valdecilla University Hospital, Santander, Spain; 2 Valdecilla virtual Hospital, Valdecilla Biomedical Research Institute (IDIVAL), Santander, Spain; 3 Department of Colorectal Surgery, University Hospitals of Derby and Burton NHS Foundation Trust, Derby, UK; 4 School of Medicine, Royal Derby Hospital, University of Nottingham, Derby, UK; 5 Department of Research and Skills Development, ORSI Academy, Melle, Belgium; 6 Section of Coloproctology, Department of Surgery, University of Erlangen-Nürnberg, FAU, Erlangen, Germany

## Abstract

**Background:**

This study aimed to evaluate the use of binary metric-based (proficiency-based progression; PBP) performance assessments and global evaluative assessment of robotic skills (GEARS) of a robotic-assisted low anterior rectal resection (RA-LAR) procedure.

**Method:**

A prospective study of video analysis of RA-LAR procedures was carried out using the PBP metrics with binary parameters previously developed, and GEARS. Recordings were collected from five novice surgeons (≤30 RA-LAR previously performed) and seven experienced surgeons (>30 RA-LAR previously performed). Two consultant colorectal surgeons were trained to be assessors in the use of PBP binary parameters to evaluate the procedure phases, surgical steps, errors, and critical errors in male and female patients and GEARS scores. Novice and experienced surgeons were categorized and assessed using PBP metrics and GEARS; mean scores obtained were compared for statistical purpose. Also, the inter-rater reliability (IRR) of these assessment tools was evaluated.

**Results:**

Twenty unedited recordings of RA-LAR procedures were blindly assessed. Overall, using PBP metric-based assessment, a subgroup of experienced surgeons made more errors (20 *versus* 16, *P* = 0.158) and critical errors (9.2 *versus* 7.8, *P* = 0.417) than the novice group, although not significantly. However, during the critical phase of RA-LAR, experienced surgeons made significantly fewer errors than the novice group (95% CI of the difference, Lower = 0.104 – Upper = 5.155, df = 11.9, t = 2.23, p = 0.042), and a similar pattern was observed for critical errors. The PBP metric and GEARS assessment tools distinguished between the objectively assessed performance of experienced and novice colorectal surgeons performing RA-LAR (total error scores with PBP metrics, *P* = 0.019–0.008; GEARS scores, *P* = 0.029–0.025). GEARS demonstrated poor IRR (mean IRR 0.49) and weaker discrimination between groups (15–41 per cent difference). PBP binary metrics demonstrated good IRR (mean 0.94) and robust discrimination particularly for total error scores (58–64 per cent).

**Conclusions:**

PBP binary metrics seem to be useful for metric-based training for surgeons learning RA-LAR procedures.

## Teaser

In this prospective, multicentre study, we objectively assessed the performance of experienced and novice colorectal surgeons performing robotic-assisted low anterior rectal resection (for rectal cancer surgery) with two assessment methods: proficient-based progressive (PBP), and GEARS. Both assessment methods discriminated between group performances, but PBP binary metrics seem to be a more useful tool in this study. PBP binary metrics revealed that some experienced surgeons performed poorly.

## Introduction

Increasing evidence has suggested intraoperative skills are associated with patient outcomes^1,2^. The European School of Coloproctology (ESC) of the European Society of Coloproctology (ESCP) was set up to improve training and benchmark standard in different colorectal procedures and to improve patient outcomes^3^. It is, therefore, vital to identify objective, transparent, evidence-based tools to enhance training and assessment. One of these methods is proficient-based progressive (PBP) training^4^. In prospective, randomized clinical trials in different specialties, the PBP method was shown to produce skillsets that are 40–60 per cent superior to traditional training approaches^5–14^ and had a positive impact on clinical outcomes^15^.

Robotic colorectal procedures have been increasingly performed, and low anterior resection (LAR) of the rectum is one of the index colorectal operations. One of the remits of the ESC is to improve training and clinical outcomes of patients from robotic-assisted (RA) LAR. The PBP metric was previously applied to RA-LAR and obtained face and content validations^4^. RA-LAR was deconstructed to smaller components—procedural phases with steps, to recognize errors and critical errors, with distinction between sexes.

To apply these metrics widely, they were used to construct validity— to establish whether they can discriminate between the objectively assessed performances of novice and experienced surgeons, and differentiate within the experienced surgeon group (experienced surgeons whose performance is scored as above the median, and experienced surgeons whose performance is scored as below the median).

The global evaluation assessment of robotic skills (GEARS) is a widely used, although not procedure-specific, validated assessment tool for grading overall technical proficiency for robotic surgery^16^. It consists of six domains, including depth perception, bimanual dexterity, efficiency, force sensitivity, robotic control, and autonomy, with a total score range between 6 and 30.

In the pursuit of the most suitable instrument to improve RA-LAR training and assessment, it would be of value to compare the objective assessment of RA-LAR using both PBP and GEARS methodologies. A comparison of PBP metrics and GEARS has not yet been reported for a robotic-assisted colorectal procedure performed on patients. On this basis, the objectives of this study were to obtain construct validity in RA-LAR using PBP binary metrics and to compare the assessment of PBP and GEARS methods to distinguish procedure-specific performance of RA-LAR.

## Methods

A prospective study of video analysis of RA-LAR was carried out using the parameters previously published^4^.

Colorectal surgeons with different degrees of experience from different European countries participated in the study, submitting unedited videos of RA-LAR. These surgeons were selected through the ESCP Colorectal Robotic Surgery Working Group and network due to their volume and quality of robotic colorectal surgery practice, and educational interests. Colorectal surgeons were categorized as ‘experienced’ if they had performed at least 30 RA-LAR procedures before sending the first unedited video, or ‘novice’ if they had performed fewer than 30. This cut-off was chosen based on learning curves for RA-LAR in the literature^17–22^. Experienced surgeons were further classified according to their scored performance (total error scores) based on PBP metrics (see statistics in following section) and these groups were used to compare the assessment instruments for data analysis.

The study was approved by the Region of Cantabria Research Ethical Committee in Santander, Cantabria, Spain.

### Assessment tools

GEARS and PBP scores were obtained for the experienced and novice groups.

The development of the PBP that characterizes a ‘reference’ approach to RA-LAR was described previously^4^. In brief, the RA-LAR was characterized by the metrics team and verified by a Delphi panel. RA-LAR was deconstructed into procedure phases and steps, with errors and critical errors defined. An example of one of the procedure phases is shown in *Fig. S1*^4^. These procedure phases, steps, errors, and critical errors are well defined, unambiguous, and either occurred or did not occur (binary).

The performance metrics consist of 14 procedure phases and 129 steps, with 88 errors and 115 critical errors in women, and 87 errors and 116 critical errors in men.

Of note, phase IX of the PBP metric describes the steps and errors related to rectal resection (from the visualization of the edge of all three robotic instruments in the pelvis until the divided rectum is placed in the abdominal cavity and is in view)^4^, thus a focus was conducted for this subset^4^.

PBP binary metrics were used to evaluate the number of procedure phases, steps completed, and the number of errors, critical errors, and total errors made by the experienced and novice groups for RA-LAR. For the critical phase of RA-LAR (phase IX), the number of steps completed, and the number of errors, critical errors, and total errors made by the groups were evaluated.

GEARS is a validated assessment tool and has been widely used for robotic surgery and described previously^16,23–25^. It consists of six domains: depth perception, bimanual dexterity, efficiency, force sensitivity, robotic control, and autonomy, with a total score range between 6 and 30.

### Assessors

Two consultant colorectal surgeons with a special interest in robotic surgery (each has performed >50 robotic colorectal resections) were trained to be assessors in this study by a senior behavioural scientist, an education-training expert, and an experienced colorectal robotic surgeon. Assessor training was described previously^26,27^. In brief, 8 h of meetings via four conference calls were conducted online using the Zoom^©^ platform (San Jose, California, USA). Both assessors studied the methods of PBP metrics for RA-LAR and GEARS scoring in detail. They then used multiple unedited videos of RA-LAR (different from those used in the study) performed by different surgeons of varying degree of expertise to illustrate the standard for scoring reliably using both the PBP and GEARS methodology. The next stage of training required each assessor to score the video independently until the inter-rater reliability (IRR; agreements /  (agreements + disagreements)) was consistently equal to or more than 0.8^26,28^. Any conflicts or uncertainty of the scoring were further discussed to improve the clarity of the assessment techniques.

Once both assessors could reliably score the videos independently with an IRR greater than or equal to 0.8, they then scored the videos selected for this investigation. Both assessors were blinded regarding the identity or level of expertise of the operating surgeon. Each video was scored by two independent assessors using both PBP and GEARS, and scores were tabulated.

### Outcome of interest

The main outcome of interest was the ability of PBP RA-LAR metrics to differentiate between experienced and novice groups. A secondary outcome of interest was the comparison of PBP metrics and GEARS methods to distinguish the procedure-specific performance of RA-LAR. Finally, the surgeons’ operative experience (defined as the number of procedures performed) was correlated with the procedure steps completed and errors as assessed by PBP and GEARS.

### Statistical analysis

The IRR was calculated between assessors using both PBP and GEARS scores. The experienced surgeon group scores were divided based on the median of the total error scores (error score + critical error score = total errors). Experienced surgeons who made the least number of total errors were classified as performing ‘below the median’ (BTM) with a lower error rate (LoErrR), and experienced surgeons who made the greatest number of total errors were classified as ‘above the median’ (ATM) with a higher error rate (HiErrR)^29,30^ (factor 1). If the surgeon provided more than one video for assessment, the repeated measure was considered as factor 2.

The PBP measures for each surgeon and recorded procedure were the total number of procedure steps completed; the total number of errors made; the total number of critical errors made; and the sum of errors and critical errors (total errors). GEARS measures were the total score for each surgeon for each video. The mean and 95 per cent confidence interval are presented in the figures for procedure steps completed, errors made, critical errors made, total errors, and GEARS score.

Statistical analysis was performed with SPSS^®^ (IBM, Armonk, New York, USA). A 2 × 2 mixed model ANOVA was used to determine to detect statistical difference for the endpoints. A Pearson product moment correlation coefficient was used to assess the strength of the relationship between surgical operative experience, performance metrics, and GEARS (procedure steps and errors).

## Results

Nine experienced colorectal surgeons submitted 13 videos and five novice colorectal surgeons submitted 7 videos (*Fig. S2*).

### PBP binary metrics

**Fig. 1:**
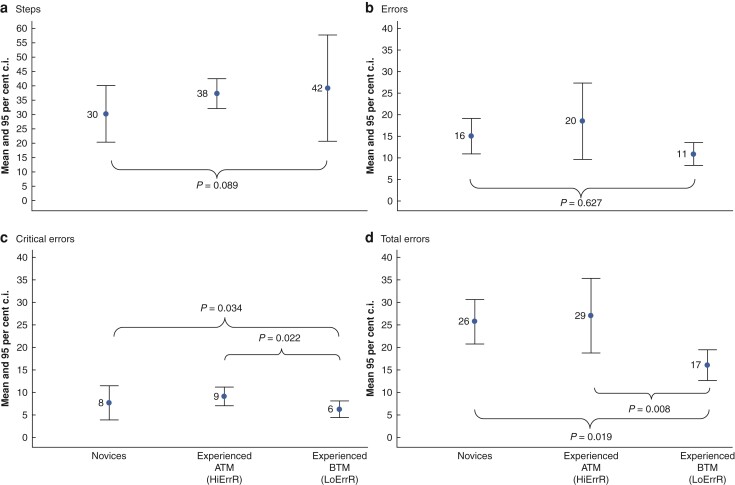
**a** **–d Mean and 95 per cent confidence interval (c.i.) for steps, errors, critical errors, and total error scores for the novice, experienced below the median (BTM), and experienced above the median (ATM) robotic surgeons’ groups for RA-LAR. RA-LAR, robotic-assisted low anterior resection; HiErrR, high error rate; LoErrR, low error rate**

The experienced group of surgeons demonstrated considerable performance variability, which was then compared with that of the novice group. *Figure 1* shows the mean and 95 per cent confidence interval for the mean procedure steps (*Fig. 1a*), procedure errors (*Fig. 1b*), critical errors (*Fig. 1c*), and total errors (*Fig. 1d*) made by the novice surgeons, experienced BTM, and experienced ATM groups.

The experienced BTM group completed more procedure steps (*Fig. 1a*) than the other two groups, but this was not statistically significant. Likewise, they made fewer procedure errors than both the novice and experienced ATM groups (*Fig. 1b*). For the error metric, the experienced ATM group made more errors than the novice or experienced BTM groups (experienced BTM mean 10.89 *versus* experienced ATM mean 20.19 *versus* novices mean 15.85). This difference was also not statistically significant for the novice group but was significant for the experienced ATM group (95 per cent c.i. 3.16 to 16.26, d.f. = 24.49, *t* = 3.056, *P* = 0.005). The experienced ATM also demonstrated the greatest performance variability (experienced BTM, 2.03 s.d. *versus* experienced ATM, 11.03 s.d. *versus* novices, 4.18 s.d.). As shown in *Fig. 1c* the experienced ATM also made the largest number of critical errors (experienced BTM mean 6.33 *versus* experienced ATM mean 9.17 *versus* novices mean 7.8). Overall, the experienced BTM group made significantly fewer critical errors than the novice group (95 per cent c.i. 0.252 to 5.335, d.f. = 11.76, *t* = 2.4, *P* = 0.034) and the experienced ATM group (95 per cent c.i. 0.547 to 6.084, d.f. = 13.87, *t* = 2.57, *P* = 0.022). The largest performance differences were observed for combined error scores (errors + critical errors). The experienced BTM group made 58 per cent fewer errors than the novice group and 64 per cent fewer than the experienced ATM group (*Fig. 1d*). Both differences were statistically significant (for the difference between the novice group 95 per cent c.i. 1.883 to 17.329, d.f. = 13.49, *t* = 2.68, *P* = 0.019 and for the difference between the experienced ATM group, 95 per cent c.i. 3.192 to 18.265, d.f. = 16.8, *t* = 3.01, *P* = 0.008).

**Fig. 2:**
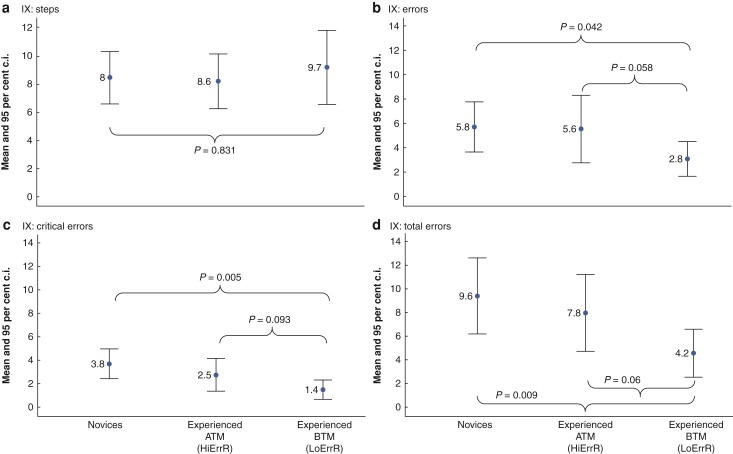
**a**–**dMean and 95 per cent confidence interval (c.i.) for steps, errors, critical errors, and total error scores for the novice, experienced below the median (BTM), and experienced above the median (ATM) robotic surgeons’ groups for phase IX of RA-LAR. RA-LAR, robotic-assisted low anterior resection; HiErrR, high error rate; LoErrR, low error rate**

The same type of analysis was also completed for the performance of the three groups on phase IX (rectal dissection/rectal transection (TME/LAR)) and the mean and 95 per cent confidence intervals for procedure steps completed, errors, critical errors, and total errors are shown in *Fig. 2a–d*. The experienced BTM group completed the most steps (*Fig. 2a*) but this was not statistically significant. They did, however, make significantly fewer errors (46 per cent) than the novice group (95 per cent c.i. 0.104 to 5.155, d.f. = 11.9, *t* = 2.23, *P* = 0.042). They also made 44 per cent fewer errors than the experienced ATM group (*Fig. 2b*) but this was not significant (95 per cent c.i. −0.095 to 5.153, d.f. = 16, *t* = 2.04, *P* = 0.058). A similar pattern was observed for critical errors. The experienced BTM group made 59 per cent fewer critical errors than the novice group and 45 per cent fewer than the experienced ATM group (*Fig. 2c*) but only the difference between the novice group was statistically significant (novice group, 95 per cent c.i. 0.76 to 3.647, d.f. = 15.1, *t* = 3.25, *P* = 0.005; experienced group, 95 per cent c.i. −0.233 to 2.718, d.f. = 16, *t* = 1.78, *P* = 0.093).

Errors and critical errors for each group were combined into a total error score, the results showed that the experienced BTM group made the fewest errors and demonstrated the greatest performance consistency as evidenced by the smallest confidence intervals (*Fig. 2d*). They made 51 per cent fewer errors than the novice group (95 per cent c.i. 1.381 to 8.323, d.f. = 14.6, *t* = 2.99, *P* = 0.009) and 43 per cent fewer errors than the experienced ATM group (95 per cent c.i. −0.165 to 7.045, d.f. = 16, *t* = 2.02, *P* = 0.06).

Assessors scoring videos submitted from novice and experienced surgeons had a mean IRR for the metric-based performance assessments of 0.94 (IRR range 0.93–0.97). None of the assessments fell below the 0.8 IRR quality threshold.

### GEARS scores

**Fig. 3:**
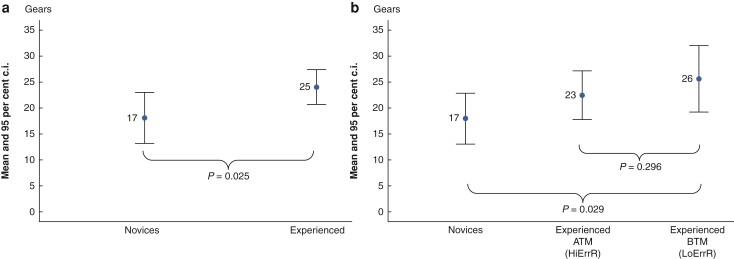
**a** **and** **b** **Mean and 95 per cent confidence interval (c.i.) for GEARS scores for the RA-LAR procedure.** **a** **Novice and experienced groups.** **b** **Novice and experienced groups below the median (BTM) and experienced above the median (ATM). RA-LAR, robotic-assisted low anterior resection; HiErrR, high error rate; LoErrR, low error rate**

*Figure 3* shows the mean and 95 per cent confidence intervals of surgeons’ operative performance using the GEARS assessment. *Figure 3a* shows the comparison between the two groups. The experienced surgeons had a 35 per cent higher rating than the novice surgeons, which was statistically significant (95 per cent c.i. 0.87 to 11.74, d.f. = 17.8, *t* = −2.44, *P* = 0.025). *Figure 3b* shows the scores of the experienced surgeons divided into ATM and BTM, as described previously. In this analysis, experienced surgeons BTM were rated as performing 41 per cent better than the novice group (95 per cent c.i. −14.12 to −0.865, d.f. = 14.55, *t* = −2.42, *P* = 0.029) and 15 per cent better than the experienced ATM group (95 per cent c.i. −9.802 to −3.177, d.f. = 16.9, *t* = −1.08, *P* = 0.299).

Assessors scoring videos submitted from novice and experienced surgeons had a mean IRR of the GEARS scores of 0.49 (range, 0.34–0.63).

### Surgeon experience and performance

Overall, the surgeon’s operative experience positively correlated with number of procedure steps completed (*r* = 0.449, *P* = 0.062) although this was not significant (novice group *r* = −0.527, *P* = 0.224 and experienced group *r* = 0.427, *P* = 0.19). The GEARS scores for all surgeons were significantly different (*r* = 0.594, *P* = 0.009) for all assessments but not for subgroups (novice group *r* = −0.472, *P* = 0.284 and experienced group *r* = 0.581, *P* = 0.061). Operative experience also correlated moderately strongly (negatively) with total errors *(r* = −0.517, *P* = 0.028) but this was only observed for the experience surgeon group in an analysis of subgroup performance (novice group *r* = −0.397, *P* = 0.378 and experienced group *r* = −0.889, *P* < 0.000).

## Discussion

This study was designed and developed with the main objectives of analysing the use of PBP binary metrics for RA-LAR and to compare PBP and GEARS methods to distinguish procedure-specific performance of a colorectal procedure. In this study, the binary-scored metrics and the GEARS assessment instrument both discriminated between the performance of experienced and novice colorectal surgeons performing RA-LAR, with an IRR for the binary-scored metrics consistently above 0.8. In contrast, none of the GEARS assessment scores was above 0.8 IRR. Despite being widely used in robotic surgery, low levels of IRR for the GEARS assessment have started to emerge in the surgical literature^31^. This finding needs to be further studied and better understood.

In contrast, the binary-scored performance metrics demonstrated good IRR across all surgeons in the novice and experienced surgeon groups. The error metrics seem to have greater sensitivity for the assessment of performance quality. This finding is emerging with reliable consistency^6,30,32^. It is now understood that a surgeon can perform all the steps and score well on this metric, but that they could perform the steps with several errors^33^. In contrast, performance errors are a better assessment of performance quality. In a systematic review and meta-analysis on prospective randomized and blinded clinical studies using PBP training methods with binary-scored metrics, performance errors emerged as the best discriminator of surgical performance across all studies (∼60 per cent difference in ratio of means)^6^.

The Institute of Medicine has argued that medicine must move to a training and assessment system that is outcome based and accurately reflects the skill level of the trainee at the assessment point^34^. This is a departure from the past where completion of a course, number of procedures carried out, or accumulated surgical experience was used as a surrogate measure of surgeon/trainee performance^35^. The skill level of the operating surgeon is emerging as a better predictor of operative performance and clinical outcomes on patients^1,2^. The emergence of this finding has not completely come as a surprise, particularly to surgeons who witnessed the roll-out and adoption of minimally invasive surgery and the observed learning curve, even for some very experienced surgeons^36^. Of some concern from the findings in this study is the finding that a small group of the experienced surgeons performed worse on all metrics than the novice group. This finding has also been reported in other specialties^25,30^.

The robotic surgical community have learned many important lessons from this evolution in surgical practice. They understand and value the lessons learned from using simulations for skills training outside the operating room^37,38^. They also understand the pre-eminence of a surgeon-derived and structured curriculum to ensure that training is more than an educational experience^5^. The effectiveness of the Arthroscopic Association of North America (AANA) curricular approach to training image-guided arthroscopic skills was primarily to do with the way that simulations (including cadaveric tissues) were configured to create a coherent and structured curriculum^39,40^.

Central to the effectiveness of the AANA curriculum were validated metrics for the assessment of performance and standardized metric-based feedback to trainees. AANA developed and validated the performance metrics for the different training models before conducting the trial^11,41–43^. Metric development and validation are relatively new to surgery but (particularly in the USA) this is a mature industry with established and agreed validation standards. These guidelines are unambiguous about these standards and clearly state that if a validated assessment is demonstrated to be unreliable (IRR greater than 0.8) it is by default not valid^25^.

The binary-scored metrics demonstrated consistently high IRR levels, discriminated between experienced and novice surgeons (evidence of construct validity), and between the experienced surgeons who were performing ATM and BTM (evidence of discriminative validity), particularly for the more serious critical errors scores and total errors. The binary metrics are also more useful for the delivery of specific, metric-based formative feedback of performance during the training of robotic surgical skills. Furthermore, the binary metrics facilitate the construction of standardized courses with quantitively defined proficiency benchmarks^44^ for robotic surgeons learning to perform RA-LAR. Every effort must be made by surgical training bodies to ensure proficient skill levels of colorectal surgeons learning to use robotic-assisted devices to perform advanced surgical procedures such as LAR. Metric-based feedback to trainees that is specific, objective, transparent, and fair is strong foundation from which to build a colorectal robotic surgery training program^45^. The main aims of this study were to obtain the construct validity for the PBP binary metrics and compare this with a readily available GEARS assessment. The limitations of this study include small sample size, and that the performance assessment of surgeons in this study is based on the assessment tools without knowing clinical outcomes. Future studies with patient outcomes will further explore the skills and outcome relationship^46^.

Binary performance metrics and GEARS assessments discriminated between the objectively assessed performance of experienced and novice surgeons who performed RA-LAR. The binary metrics demonstrated greater discrimination between surgeon performance than GEARS. GEARS assessments demonstrated poor levels of IRR. Good levels of assessment reliability are an imperative attribute for valid assessment tools. These results indicate that binary metrics are probably more useful for metric-based formative feedback during the training of colorectal surgeons learning RA-LAR procedures.

## Supplementary Material

zrac041_Supplementary_Data

## Data Availability

The data that support the findings of this study are available from the corresponding author (S.T.) upon reasonable request.
