# Supplementary figures and images for: Intraoperative robotic-assisted low anterior rectal resection performance assessment using procedure-specific binary metrics and a global rating scale
Source: BJS Open. 2022 May 11;6(3):zrac041. doi: 10.1093/bjsopen/zrac041 (PMC9092445; doi:10.1093/bjsopen/zrac041)

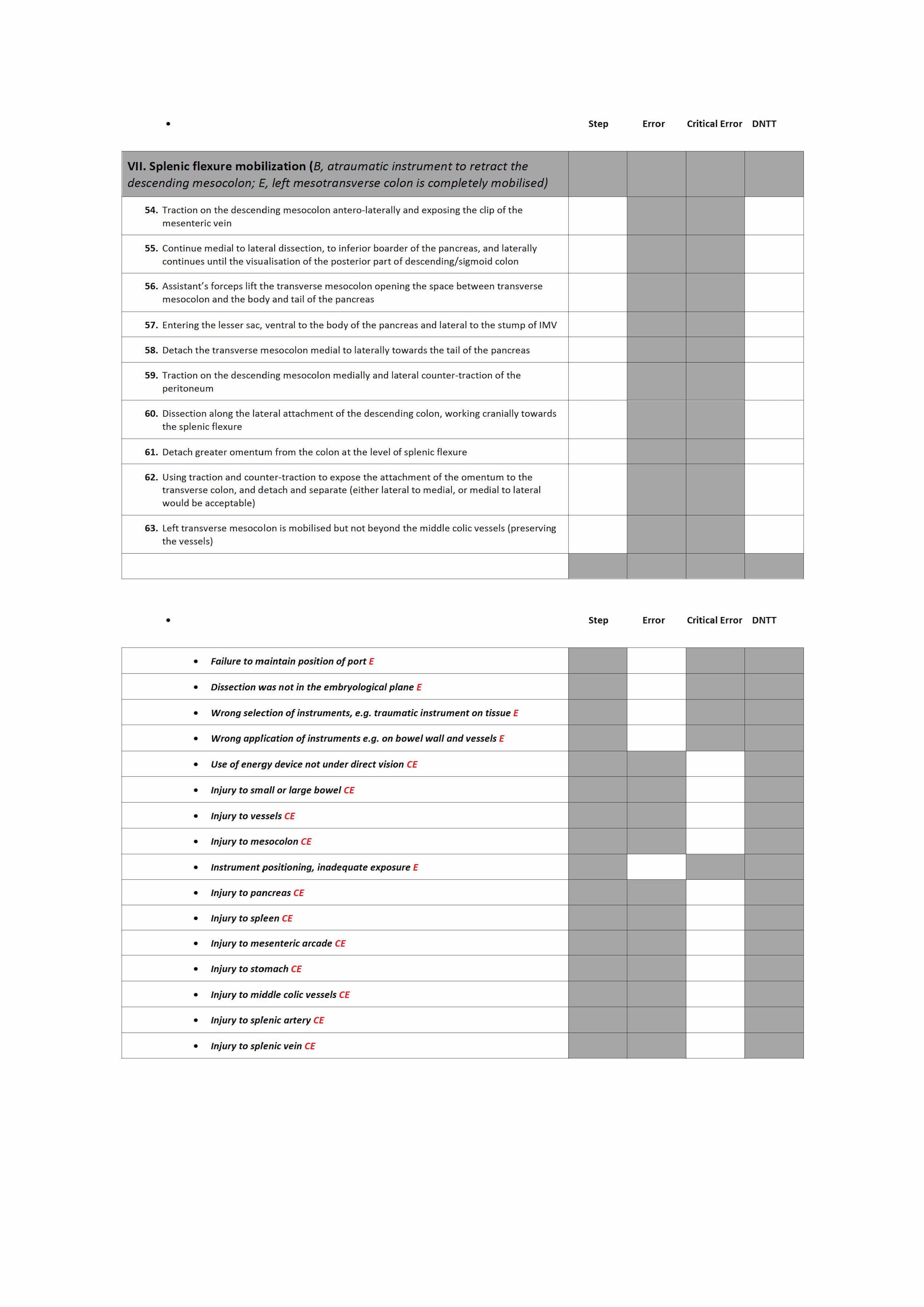

Supplement: zrac041_Supplementary_Data [file zrac041_supplementary_data.zip › Supplementary_Figure_1.jpg]

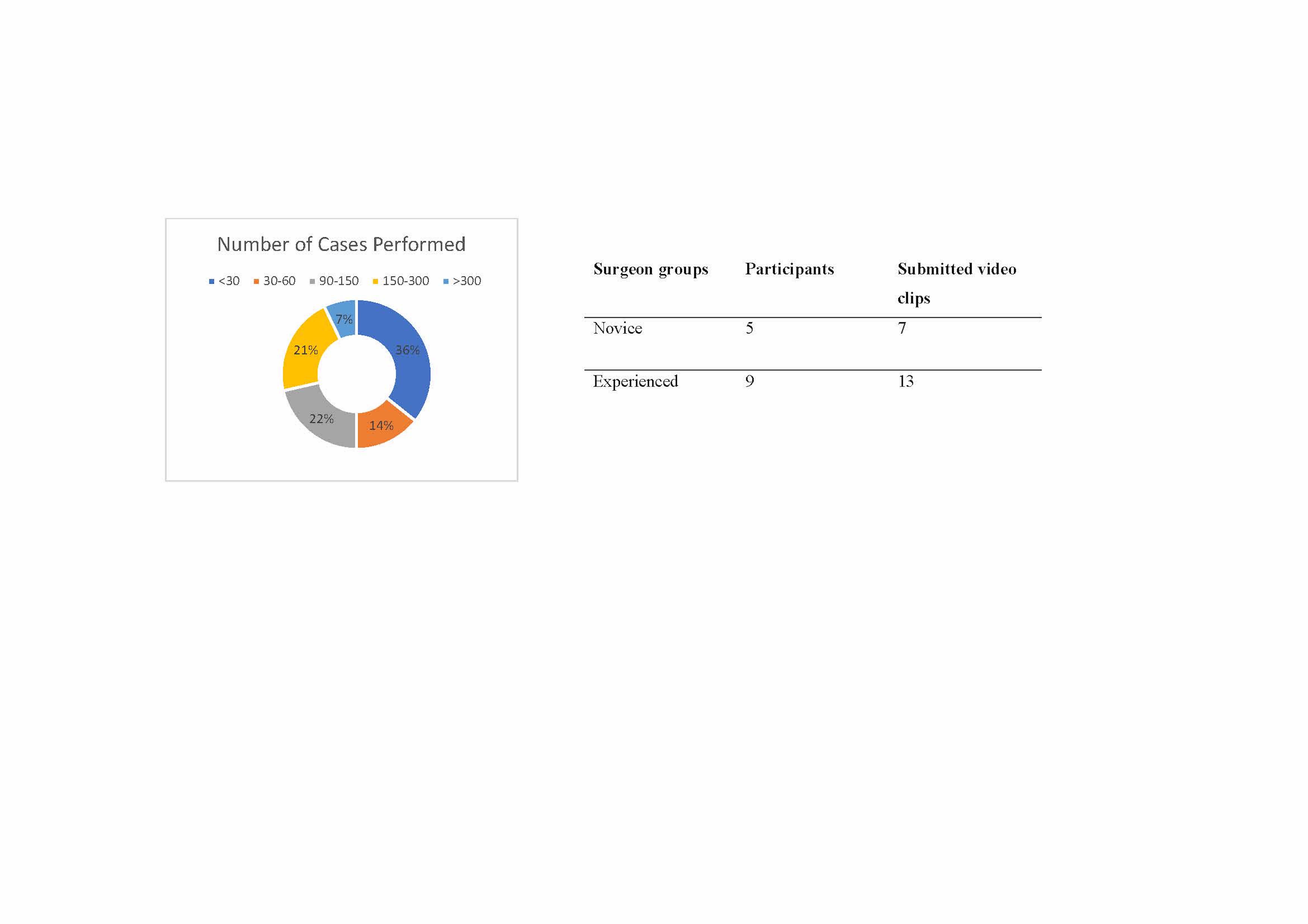

Supplement: zrac041_Supplementary_Data [file zrac041_supplementary_data.zip › Supplementary_Figure_2.jpg]
